# Isoaspartyl Formation in Creatine Kinase B Is Associated with Loss of Enzymatic Activity; Implications for the Linkage of Isoaspartate Accumulation and Neurological Dysfunction in the PIMT Knockout Mouse

**DOI:** 10.1371/journal.pone.0100622

**Published:** 2014-06-23

**Authors:** Aleksandra Dimitrijevic, Zhenxia Qin, Dana W. Aswad

**Affiliations:** Department of Molecular Biology & Biochemistry, University of California Irvine, Irvine, California, United States of America; University of South Florida College of Medicine, United States of America

## Abstract

Isoaspartate (isoAsp) formation is a common type of spontaneous protein damage that is normally kept in check by the repair enzyme protein-L-isoaspartyl methyltransferase (PIMT). PIMT-KO (knockout) mice exhibit a pronounced neuropathology highlighted by death from an epileptic seizure at 30 to 60 days after birth. The mechanisms by which isoaspartyl damage disrupts normal brain function are incompletely understood. Proteomic analysis of the PIMT-KO mouse brain has shown that a number of key neuronal proteins accumulate high levels of isoAsp, but the extent to which their cellular functions is altered has yet to be determined. One of the major neuronal targets of PIMT is creatine kinase B (CKB), a well-characterized enzyme whose activity is relatively easy to assay. We show here that (1) the specific activity of CKB is significantly reduced in the brains of PIMT-deficient mice, (2) that *in vitro* aging of recombinant CKB results in significant accumulation of isoAsp sites with concomitant loss of enzymatic activity, and (3) that incubation of *in vitro* aged CKB with PIMT and its methyl donor S-adenosyl-L-methionine substantially repairs the aged CKB with regard to both its isoAsp content and its enzymatic activity. These results, combined with similarity in phenotypes of PIMT-KO and CKB-KO mice, suggests that loss of normal CKB structure and function contributes to the mechanisms by which isoAsp accumulation leads to CNS dysfunction in the PIMT-KO mouse.

## Introduction

Creatine kinases (EC 2.7.3.2) are a family of enzymes that catalyze reversible transfer of the phosphate group between ATP and creatine, playing a crucial role in intracellular energy metabolism of cells with high and fluctuating energy requirements [1]–[3]. There are four major isozymes of creatine kinase that are products of separate genes; two cytosolic forms, predominant in brain (CKB) or muscle (CKM), and two mitochondrial forms (the ubiquitous and sarcomeric CKs). CKB is essential for optimal performance of central nervous system (CNS) function, and defects in creatine metabolism have been associated with a wide range of neuropathology in humans [4]. Knockout of the CKB gene in mice results in a phenotype with impaired learning, diminished habituation, abnormal microstructure in the hippocampus, and increased susceptibility to epileptic seizures [5]. Deficiency of CKB activity in human neurological disease has been attributed mainly to oxidative damage [6]–[8].

**Figure 1:**
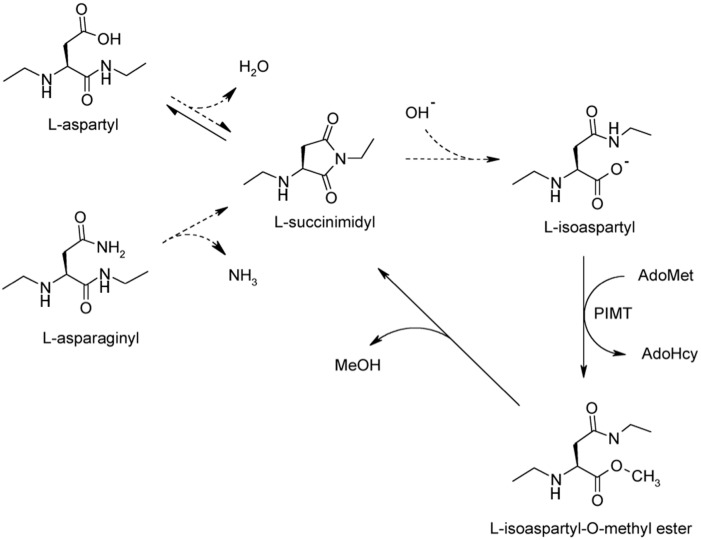
Mechanism of isoaspartate formation and PIMT-catalyzed repair. Under physiological conditions, deamidation of asparagine residues or dehydration of aspartic acid residues results in the formation of a metastable intermediate succinimide which spontaneously hydrolyzes to form a mixture of normal L-aspartyl and atypical L-isoaspartyl linkages. PIMT, using AdoMet as a methyl donor, selectively methylates the isoaspartyl α-carboxyl group to form a highly labile methyl ester. Spontaneous demethylation occurs within minutes to reform the original succinimide, with release of methanol as a by-product. This succinimide is now the starting point for further cycles of repair, resulting in near complete conversion of the isoaspartyl β-linkages to normal aspartyl α-linkages.

In 2006 we reported a proteomics study suggesting that CKB is highly susceptible to spontaneous formation of abnormal isoaspartyl (isoAsp) residues *in vivo* [9]. Formation of isoAsp is considered to be a deleterious modification that can significantly impact protein activity and elicit autoimmunity [10]–[14]. IsoAsp arises from deamidation of asparagine or dehydration of aspartic acid, leading to formation of a metastable succinimide (cyclic imide) intermediate that hydrolyzes to a mixture of aspartyl and isoaspartyl linkages (Fig. 1). IsoAsp formation occurs most readily at sequences in which the side chain of the C-flanking amino acid is relatively small and hydrophilic. Asx-Gly, Asx-Ser and Asx-His sequences, particularly when located in highly flexible protein regions, constitute “hot spots” for isoAsp formation. Cellular mechanisms for dealing with isoaspartyl protein damage include urinary excretion of the damaged proteins [15], degradation by isoAsp-selective proteases [16], [17], and enzymatic repair. Regarding the latter mechanism, isoAsp residues in peptides and proteins are specifically recognized and repaired by the action of protein L-isoaspartyl O-methyltransferase (PIMT, EC 2.1.1.77) [18]–[23]. PIMT utilizes the cofactor S-adenosyl-L-methionine (AdoMet) to transfer a methyl group onto the α-carboxyl group of isoAsp sites leading to succinimidyl intermediate that subsequently hydrolyze to L-Asp and L-isoAsp (Fig. 1). Continuing cycles of PIMT action efficiently repair L-isoAsp sites *in vitro* [23]–[25], while reduction of PIMT activity in cultured cells or knockout (KO) mice dramatically increases the level of isoAsp-containing proteins [26]–[29]. PIMT is widely distributed in mammalian tissues, but is particularly rich in the CNS [30]–[33]. The critical need for PIMT in brain is evident from the overt neurological phenotype of PIMT-deficient mice; increased brain size, abnormal neuro-anatomical and electrophysiological properties of hippocampal cells along with reduced cognitive function [34], atypical open-field behavior [35], and fatal epileptic seizures beginning at 4 weeks of age [27], [28]. A proteomic study utilizing the PIMT-KO mouse revealed CKB as one of 22 major targets for PIMT in the brain [9].

There is considerable interest in how isoAsp formation leads to disruption of CNS function. Individual variation in PIMT activity has been linked to successful aging in humans and resistance to heat and metabolic stress in model organisms such as *D. melanogaster and C. elegans* [36]–[38]. Given the phenotype of the PIMT-KO mouse, extreme deficits in PIMT in humans would likely lead to neurodevelopmental disorders of the young, while moderate deficits might lead to an acceleration of age-related decline in CNS function. The purpose of the current study was to corroborate the identity of CKB as a physiological target of PIMT and determine if CKB catalytic function is altered by isoAsp formation. We report here that CKB activity is significantly decreased in parallel with its accumulation of isoAsp sites both *in vivo* and *in vitro*. Moreover, the isoAsp accumulation and activity loss can be substantially reversed *in vitro* with PIMT. The significance of these findings is enhanced by noting the marked similarity in phenotypes of the PIMT-KO and CKB-KO mice.

## Material and Methods

### Reagents

S-Adenosyl-[methyl-3H]-L-methionine (3H-AdoMet) was purchased from PerkinElmer Life Sciences. A rabbit polyclonal antibody raised against full-length human recombinant CKB was purchased from Abnova (#PAB19125). A monoclonal rabbit antibody to β-actin (used as a loading control) was from Cell Signaling (#4970). Secondary antibody (horse radish peroxidase-linked donkey anti-rabbit IgG) and Pierce ECL Plus Western Blotting Substrate were purchased from GE/Amersham and Thermo/Pierce, respectively. Recombinant rat PIMT was expressed in *E. coli* and purified as described previously [9]. Recombinant human CKB was purchased from MyBioSource. Isoaspartyl delta-sleep inducing peptide (WAGGD∧ASGE; where ∧ designates an isopeptide bond) was purchased from Bachem.

### Mice

PIMT^+/−^ (HZ) founder mice were a gift of Dr. Mark Mamula (Yale University, New Haven, CT) and were originally generated by inserting a neo cassette into exon one of the pcmt1 gene [27]. PIMT^−/−^ (KO) and PIMT^+/+^ (WT) C57BL/6 mice were obtained by intercrossing PIMT-HZ mice. Genotyping was determined by tail DNA PCR analysis recognizing both the neo cassette and pcmt1 gene (Transnetyx, Inc., Cordova, TN). All animal housing and procedures were performed using a protocol approved by the Institutional Animal Care and Use Committee of the University of California, Irvine. Mice were anesthetized with a lethal dose of Euthasol prior to decapitation at 4 weeks of age.

### Preparation of Mouse Brain Extracts

Mouse brains were weighed immediately after removal and suspended in 9 vol of ice-cold homogenization buffer (10% (w/v) sucrose, 5 mM K-Hepes, pH 7.6, 0.5 mM EDTA, 0.1 mM DTT (dithiothreitol), 50 mM NaF and 1 mM Na_3_VO_4_ as phosphatase inhibitors, and % (v/v) mammalian protease inhibitor mixture (Sigma). The suspension was homogenized on ice using a Potter-Elvejhem tissue homogenizer and then centrifuged at 800× g for 30 min. The supernatant was centrifuged at 20,000× g for 1 h to pellet the mitochondrial CK fraction [39]. The final supernatant (hereafter ‘‘brain extract’’) was stored in aliquots at −80°C, and used as needed for determination of cytosolic CKB specific activity and assessment of CKB expression by Western blotting.

### Protein Concentration and Enzyme Activity

Protein concentration was determined using the Pierce BCA microplate assay with bovine serum albumin as a standard. CKB activity in brain extracts was determined in the direction of ATP synthesis with a commercial kit (Pointe Scientific, Inc.) that uses the coupled enzyme assay shown in scheme 1.

*Reaction 1 (CK catalyzed): creatine phosphate + Mg-ADP > ATP + creatine*

*Reaction 2 (HK catalyzed): ATP + glucose > ADP + G-6-P*

*Reaction 3 (G6PDH catalyzed): G-6-P + NADP^+^ >6-GP + NADPH + H^+^*

*CK, creatine kinase; HK, hexokinase; G6PDH, glucose 6-phosphate dehydrogenase; G-6-P, glucose-6-phosphate; 6-GP, 6-phosphoglucono-lactone. One unit of CKB activity is defined here as one µmol of NADPH generated per min at 37°C under initial rate conditions.*

### SDS-Polyacrylamide Gel Electrophoresis and Western Blotting

Brain extracts were subjected to SDS-PAGE on NuPAGE 4–12% gradient gels with MES running buffer, and gels were stained using a Colloidal Blue Staining Kit (all from Novex, Life Technologies). Proteins were transferred to polyvinylidene difluoride membranes (0.45 µm pore) by semi-dry electroblotting. These were incubated for 2 h at room temperature with primary antibody to CKB (1:500,000) or β-actin (1:10,000), followed by 1 h in secondary antibody (1:15,000). The chemiluminescent signals were recorded with a Nikon D700 camera and quantitated using NIH ImageJ [40].

### In Vitro CKB Aging and IsoAsp Quantitation m

Recombinant human CKB (0.4 mg/ml) was incubated in a closed microfuge tube for 16 days at 37°C in aging buffer (20 mM Tris-HCl, pH 7.5, 20 mM NaCl, 1 mM EDTA, 2% (v/v) glycerol, 0.05% (w/v) NaN_3_). Measurement of isoaspartate was determined by a methanol diffusion assay [41], [42]. In brief, proteins (100 pmol in a final volume of 50 µl) were incubated in methylation reaction buffer (100 mM sodium phosphate, pH 6.8, 4 mM recombinant rat PIMT, 100 µM [^3^H]AdoMet (500 dpm/pmol), 5 mM EDTA and 0.2 mg/ml BSA) for 30 min at 30°C. The reaction was terminated by addition of an equal volume of stop solution (400 mM Na-borate pH 10.4, 4% SDS, 2% methanol), then 50% of the sample was transferred to an accordion-pleated filter paper lodged in a Titeseal cap. Immediately the cap was fitted to a shell vial containing 2.5 ml of Liquiscint (National Diagnostics), and incubated for 1 h at 40°C prior to liquid scintillation counting. Isoaspartyl delta-sleep inducing peptide (50 pmol) was used as an internal standard for isoAsp quantitation. Optimization experiments have demonstrated that the conditions specified above result in quantitative methylation of the isoaspartyl peptide standard as judged both by ^3^H-methanol recovery and by high-performance liquid chromatography [43].

Because isoAsp quantitation was carried out on intact CKB, isoAsp sites that are buried in the normally folded protein would not be detected. Buried sites can be measured if a protein is first rendered into peptide fragments by protease treatment [44], [45]; but this was deemed unnecessary as this study is focused on the status of isoAsp sites that are accessible to PIMT-dependent repair. Moreover, previous studies with aged recombinant protein pharmaceuticals indicate that proteolytic digestion generally results in only a modest increase in measurable isoAsp sites [45]. This is consistent with the idea that isoAsp forms preferentially in flexible regions of a protein, and that such regions are typically found on the surface of a protein with good solvent exposure.

CKB specific activity was determined as described above for brain extracts.

### In Vitro Repair of Isoaspartyl Sites

Repair reactions were carried out for 6 h at 37°C as described previously [23], [46] in Pierce Slide-A-Lyzer microdialysis tubes (7-kDa cutoff) in a final volume of 50 µl. After final dialysis, CKB activity and isoAsp content were determined as described above. Methylation reactions used for repair are much longer in duration (6 h) than methylation reactions used for isoAsp quantitation (30 min), because the former requires multiple cycles of methylation/demethylation due to the inefficient yield of normal aspartyl peptide product when the succinimide hydrolyzes (Fig. 1).

### Statistical Evaluation

Results are expressed as means ± standard deviations (S.D.) for the number (*n*) of samples. Statistical significance was calculated using the Student's *t*-test. Differences between means were considered significant if *p*-values were less than 0.05.

## Results

### CKB Activity is Reduced in PIMT-KO Mice

**Figure 2:**
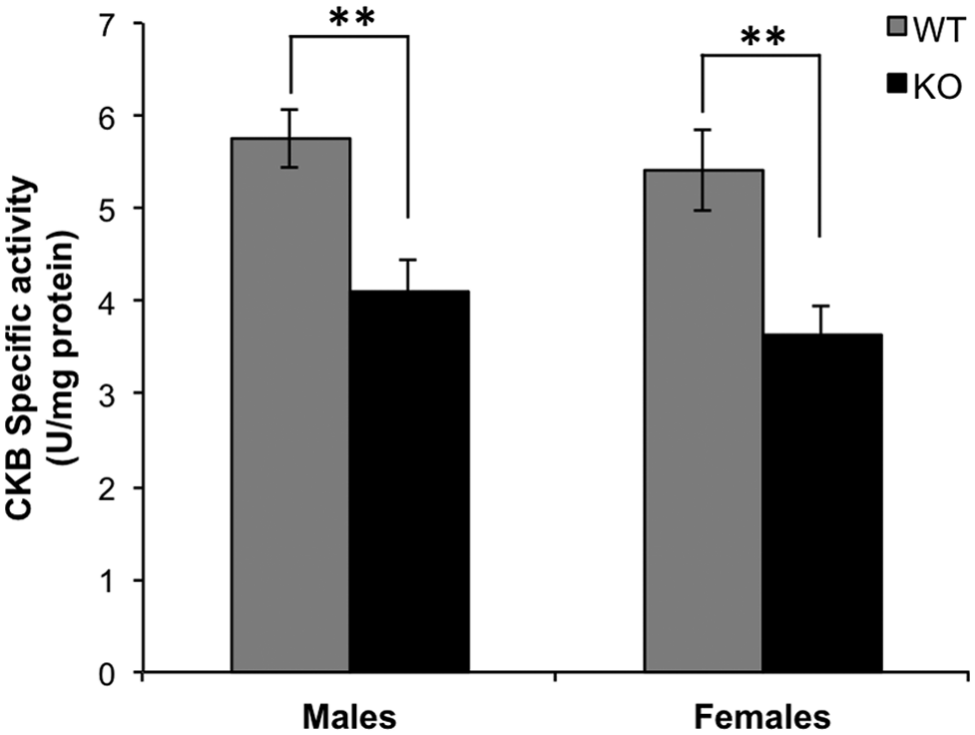
Determination of CKB specific activity in brain extracts from 4-week mice. CKB activity and protein concentrations were determined as described in Materials and Methods. Data are expressed as means ± S.D (n = 5 for each genotype). **P<0.01.

A previous study from our laboratory revealed that CKB appears to be a major substrate for PIMT in mouse brain [9]. To assess the effect of *in vivo* isoAsp accumulation on CKB function, the specific activity of CKB was determined in cytosolic extracts of PIMT-WT and -KO mouse brain (Fig. 2). The absence of PIMT reduced CKB activity by 28.9% in male mice, and by 32.9% in females. We tested both sexes because we recently found very significant differences in male vs. female mouse brains with regard to the phosphorylation of synapsin 1 (another major target for PIMT) [47], and because recent findings by others have demonstrated that sex differences in brain chemistry are pervasive and unpredictable [48]. The mean CKB activity in females was lower than in males (6.0% for WT and 11.3% for KO), but the significance was low (p>0.1).

We also compared CKB activity in mice that were WT vs. HZ with regard to the PIMT gene. PIMT activity in HZ brain extracts is 50–55% of that found in the WT, but HZ mice live a normal life span and exhibit no obvious phenotype. Using female mice only, we found the average CKB activity in HZ mice was 8% lower than in WT, but again the results did not reach statistical significance.

### Expression Levels of CKB in PIMT-WT, -HZ, and -KO Mice

**Figure 3:**
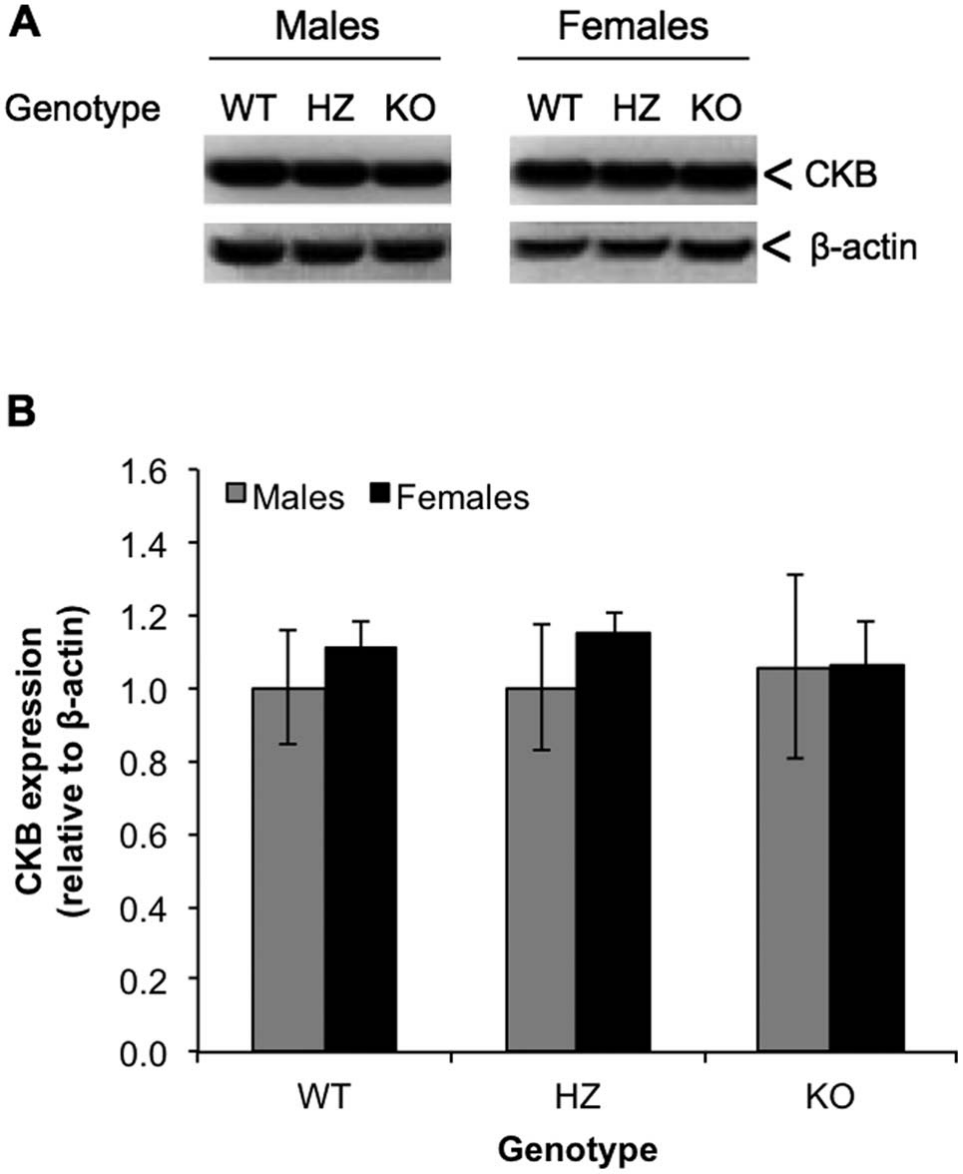
Expression of CKB in brain extracts is unaffected by PIMT genotype. (A). Representative Western blot of PIMT and β-actin expression in brains extracts from PIMT WT, HZ and KO mice. (B) Western blot quantitation after normalization of CKB bands to β-actin bands from the same sample. Data are expressed as means ± S.D (n = 5 for each genotype) relative to the WT male samples.

To determine if the reduced CKB activity observed above in the PIMT-KO mice might be due to differential expression, we assessed levels of CKB protein in PIMT-WT, -HZ, and -KO male mice by Western blot using a polyclonal antibody to full-length human recombinant CKB. Because of the similar molecular weights of CKB (42.6) and β-actin (41.7 kDa; the loading control), the same samples were analyzed separately for β-actin in parallel Western blots. The results show that CKB is equally expressed in the brains of all three genotypes (Fig. 3). We conclude that the reduced CKB activity observed in the PIMT-KO mice is likely the result of CKB damage arising as a consequence of isoAsp formation.

### Susceptibility of Recombinant Human CKB to In Vitro Aging

**Figure 4:**
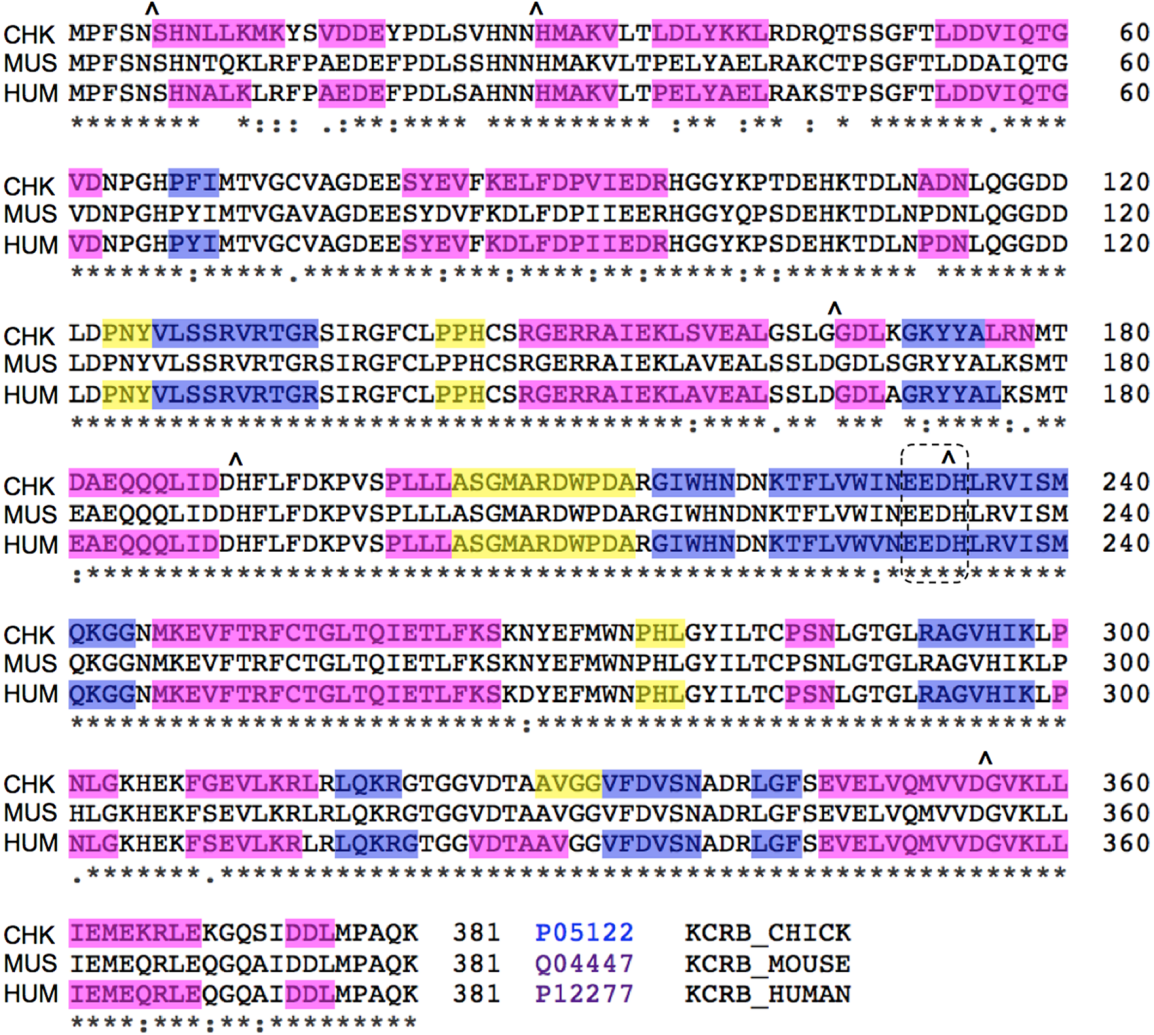
Sequence alignment of chicken (CHK); mouse (MUS) and human (HUM) CKB. Six canonical hotspots for isoAsp formation are indicated with carets (∧). Color-coding indicates regions of β-sheet (blue), α-helix (magenta), and turns (yellow) as deduced from crystal structure data according to the ExPASY (www.expasy.org) alignment application. Mouse is not color-coded because there is no crystal structure for mouse CKB. The dashed box encloses the acidic EED triplet essential for enzymatic activity.

**Figure 5:**
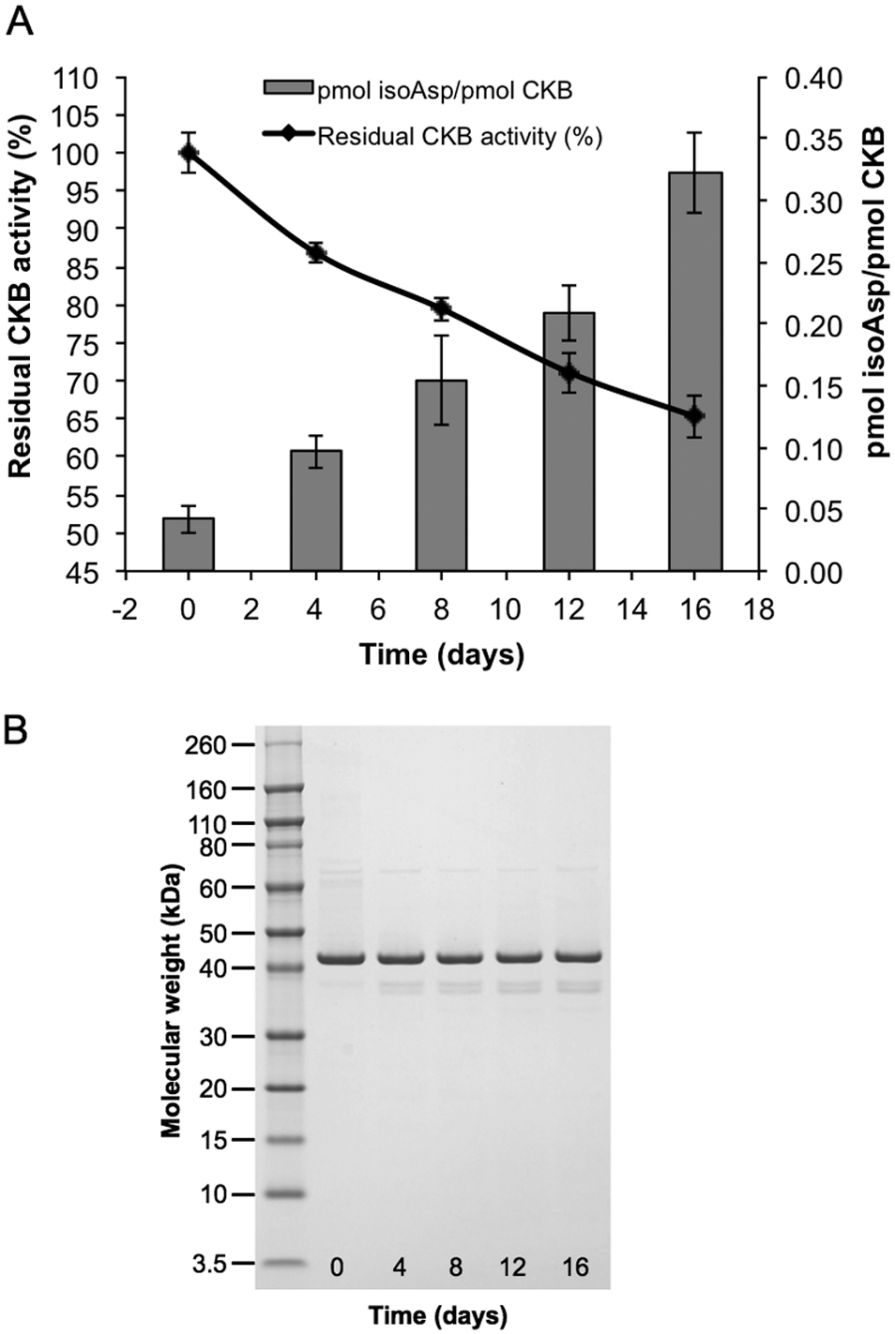
Effect of *in vitro* aging on enzymatic activity and isoaspartate content of recombinant human CKB. Recombinant human CKB (0.4 mg/mL) was aged *in vitro* at pH 7.5 and 37°C for 16 days. (A) At four-day intervals, samples were taken to assay CKB activity and isoAsp levels. Assays were performed in triplicate with standard deviations indicated by error bars. (B) SDS-PAGE of *in vitro* aged human recombinant CKB showing lack of significant degradation or aggregation.

IsoAsp formation is an autocatalytic event (Fig. 1) that appears to go through the same succinimide mechanism both *in vitro* and *in vivo* [49]. Our observation of reduced CKB activity in the PIMT-KO mouse thus predicts that purified CKB should, during *in vitro* aging at physiological pH and temperature, undergo measurable isoAsp accumulation with concomitant loss of enzymatic activity. We therefore subjected human recombinant CKB (which is commercially available and shares high sequence homology to mouse CKB; Fig. 4) to *in vitro* aging for 16 days at pH 7.5 and 37°C and then assayed for both isoAsp content and enzyme activity (Fig. 5A). CKB accumulated high levels of isoAsp (0.33 mol isoAsp/mol CKB), which was accompanied by a 34% loss of initial enzyme activity. To look for possible aggregation or proteolytic degradation that would complicate interpretation of these results, the aged samples were subjected to SDS-PAGE and subsequent staining with Coomassie Colloidal Blue (Fig. 5B). Staining revealed the expected dominant band at 43 kDa (CKB), a faint band at ∼70 kDa that did not change over time, and a pair of faint bands at ∼38 kDa whose intensity increased slightly over time. The near constant intensity of the CKB band at 43 kDa indicates that the enzyme remains substantially intact over the aging period, supporting the conclusion that loss of activity is due to mainly isoAsp formation, and not degradation or aggregation of intact CKB.

### PIMT Repairs IsoAsp Sites in Aged Recombinant CKB

**Figure 6:**
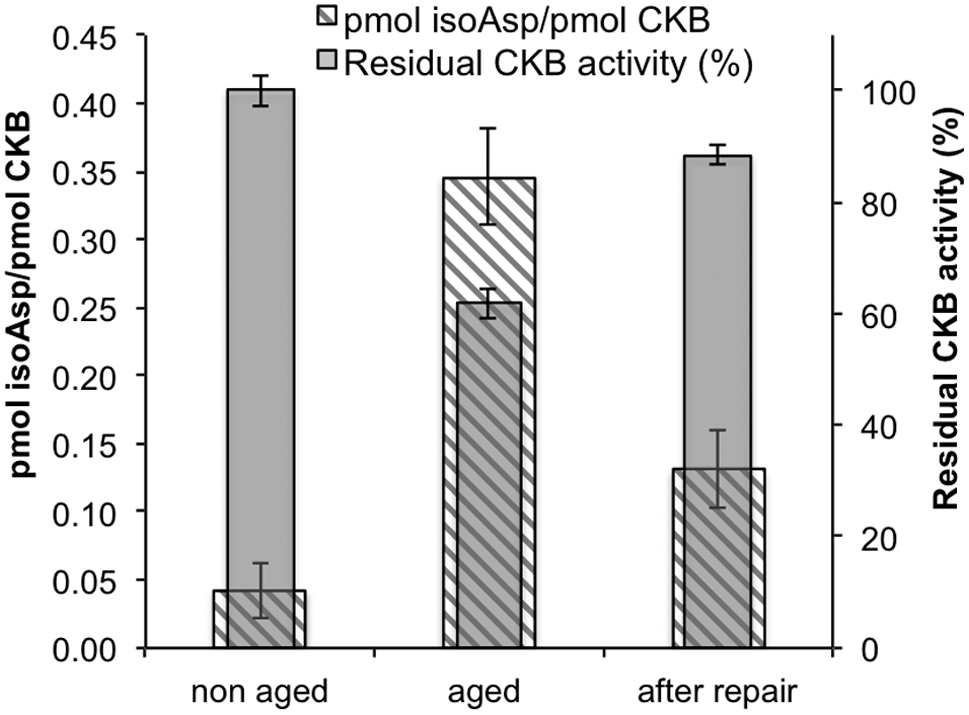
*In vitro* repair of the isoaspartyl sites in aged recombinant CKB. Aged recombinant human CKB was subjected to *in vitro* repair as described under “Materials and Methods” in the presence of PIMT and unlabeled AdoMet. CKB activity (right axis) before aging, after aging and after repair was determined and isoAsp content (left axis) was measured by a methanol diffusion assay. Each assay was performed in triplicate with error bars representing standard deviation.

Several previous studies have shown that purified PIMT catalyzes the conversion of isoaspartyl peptide bonds to normal α-carboxyl-linked peptide bonds in peptides and proteins. We tested the potential of PIMT to repair isoAsp sites and restore activity in recombinant CKB that had been subjected to *in vitro* aging for 16 days as described above. To do this we used a protocol in which the age-damaged protein is incubated for 6 h with PIMT and unlabeled AdoMet while being dialyzed against a relatively large volume of AdoMet [23], [46]. This dialysis procedure facilitates repair by constantly replenishing the AdoMet methyl donor, while preventing the accumulation of its by-product, S-adenosyl-L-homocysteine (a potent inhibitor of PIMT). After further dialysis to remove unlabeled AdoMet, portions of the analyte (in this case CKB) are methylated with ^3^H-AdoMet to assess its isoAsp content, and also assayed for enzymatic activity. As shown in Fig. 6, we found that the repair reaction restored approximately 69% of the CKB enzyme activity that was lost during aging, while eliminating approximately 57% of the isoAsp sites that had accumulated during aging.

## Discussion

Creatine kinase B is highly susceptible to isoAsp formation, as demonstrated by proteomic analysis of major methyl-accepting substrates for PIMT in PIMT-KO mouse brain extracts. The phenotype of this KO mouse includes learning deficits, lack of normal habituation, and fatal epileptic seizures at 4–8 weeks after birth. Our goal in the present study was to determine if isoAsp damage to CKB might contribute to the neuropathology resulting from PIMT deficiency. IsoAsp accumulation *per se* does not always lead to loss of biological function, as exemplified by the case recombinant human growth hormone [50], so it is of interest to determine the extent to which major *in vivo* targets of the PIMT repair enzyme actually undergo reduced biological function due to isoAsp formation. The data we present here strongly suggest that isoAsp formation in mouse CKB dramatically reduces its enzymatic function. The association between isoAsp levels and reduced activity was demonstrated with isoAsp formation occurring both *in vivo* and *in vitro*. Moreover, we were able to demonstrate that incubation of isoaspartyl-damaged CKB regains significant function when incubated with PIMT and its methyl donor AdoMet. Overall, these results suggest that CKB functional damage contributes to the neuropathology characteristic of the PIMT-KO mouse.

### PIMT-KO mice and CKB-KO mice have overlapping phenotypes

Similarities between PIMT-KO and CKB-KO mice further supports the idea that decreased CKB function contributes to the PIMT-KO phenotype. Table 1 compares the phenotypic analysis of CKB-KO mice carried out by Jost et al. [5] with corresponding studies carried out by various researchers on the PIMT-KO mouse. In short, these mice are remarkably similar with regard to (a) thigmotaxis (atypical persistent walking around the perimeter of a novel enclosure, signifying reduced habituation), (b) decreased learning in the Morris water maze test, (c) enhanced susceptibility to epileptic seizures, (d) abnormal histology in the hippocampus, and (e) normal (CKB-KO) or supra-normal (PIMT-KO) performance in the accelerating rotarod coordination test.

**Table 1:** Comparison of phenotypes of PIMT-KO and CKB-KO mice, relative to wild type mice.

Characteristic	PIMT-KO	CKB-KO^a^
Habituation: thigmotaxis^b^	“hyperactivity in the open-field test” and “a strong thigmotaxic movement pattern” (Vitali and Clarke, 2004)	“diminished open-field habituation” and “none of the (KO) mice created a home-base”
Learning: Morris water maze	“impaired spatial memory” (Ikegaya et al., 2001)	“slower to learn, but acquired the spatial task”
Epileptic seizures	Highly susceptible. Spontaneous fatal seizures at 1–3 mo. (Kim et al., 1997)	In response to pentylentetrazole, the KO mice “showed significantly more myoclonic jerks preceding the first seizure…and between the first and second seizures.”
Coordination: accelerating rotarod	“perform significantly better than their heterozygous and wild type litter-mates' (Vitali and Clarke, 2004)	“did not differ from wild type mice in their motor coordination and balance on the rotarod”
Hippocampal histology	“increased cell proliferation and granule cell number in the dendate gyrus” (Farrar et al., 2005)	“intra- and infra-pyramidyl mossy fiber field is larger in (KO) mice”

a All of these entries are from Jost et al., 2002.

b A taxis in which contact, especially with a solid body, is the directive factor.

Overall, the CKB-KO phenotype is milder than the PIMT-KO phenotype. Susceptibility to seizures is much lower in the CKB-KOs, and they live a normal life span. This difference in phenotypic severity is expected as numerous proteins (probably 50 or more) are significantly affected in the PIMT-KO. Another major *in vivo* target of PIMT, synapsin I, shows evidence of functional deficits as evidenced by its *in vivo* state of hyper-phosphorylation relative to the PIMT-WT mouse [47]. Moreover, the synapsin-KO mouse also has a phenotype (learning deficits and increased susceptibility to seizures) that overlaps with the PIMT-KO mouse.

### CKB contains several predicted hotspots for isoAsp formation

The sequences of mouse and human CKB (Fig. 4) reveal that each has six potential “hotspots” for isoAsp formation; Asx residues followed by Gly, Ser, or His. At physiological pH and temperature, isoAsp forms most rapidly at these sequences in short synthetic peptides, and is often found in age-damaged proteins, especially when these sequences occur in flexible domains. Of these six hotspots in CKB, four are located at the border of an α-helix. Asp-190 is at the trailing edge of an α-helix (181–190) located on the surface of the molecule, in one of three flexible regions that undergo large conformational changes during catalysis [51]. Asp-233 forms a hydrogen bond with the backbone amide of Thr-282, fixing the shape and stereochemistry of the active site, and is close to Arg-236, another residue essential for catalysis.

Phosphorylation is a common regulatory modification in proteins. A comprehensive survey of the human phospho-proteome has revealed that creatine kinase B has four phosphorylation sites; Ser-4, Ser-6, Thr-35, and Ser-163 [52]. The exact function of these modification sites has yet to be determined, but it is interesting to note that one of the putative hotspots for isoAsp formation in CKB (Asn-5) is sandwiched between two of these phosphorylation sites. How phosphorylation and isoAsp formation might affect each other at this locus in CKB is an attractive question for future research.

### IsoAsp formation may promote in vivo oxidation of, and autoimmunity to, CKB

CKB is prone to oxidative damage *in vivo* and can also serve as an autoantigen [53], [54]. A number of recent studies suggest oxidation and isoaspartate formation interact synergistically. Oxidation of purified hemoglobin with acetylphenylhydrazine produces a rapid increase in its isoAsp content [55], and exposure of erythrocytes to hydrogen peroxide has been found to induce isoAsp formation in membrane proteins [56]. UVA radiation has been found to trigger isoAsp formation in melanoma cells, while natural antioxidants such as hydroxytyrosol (found in olive oil) protect melanoma cells against both oxidative damage and isoAsp accumulation [57]. Oxidation of ceruloplasmin promotes conversion of asparagine to isoAsp in its two Asn-Gly-Arg (NGR) sites, thereby creating a motif (isoDGR) that can recognize the RGD-binding site of integrins [58], [59]. Cellular levels of PIMT can greatly affect the susceptibility of cells to apoptosis induced by oxidative stress, as observed in cultured human endothelial cells and the nematode *C. elegans* [38], [60]. This synergism between two common forms of protein damage suggests a potential role for isoAsp formation in oxidation of CKB *in vivo.* We note here that the histidines adjacent to Asn-28 and Asp-190 are susceptible to oxidative modification by 4-hydroxy-2-nonenal, a byproduct of peroxidation that is increased in Alzheimer's disease [61]. The structural basis for synergism between oxidation and isoAsp formation probably results from the fact that either type of damage can destabilize protein structure, which in turn can render a protein susceptible to further damage by the other mechanism.

A survey of retinal autoantigens associated with endogenous uveitis identified CKB, β-tubulin, β-actin, aspartate aminotransferase, and the voltage-dependent anion-selective channel protein as major contributors [62]. Interestingly, all four of these proteins are among the 22 polypeptides we detected as major endogenous substrates for PIMT in the KO mouse brain. Mamula and colleagues have shown that the presence of isoAsp can greatly increase a protein's ability to break tolerance in the innate immune system [14], [63]. Thus, it appears that isoAsp formation can have multiple deleterious effects on CKB: loss of catalytic activity, increased susceptibility to oxidation, and conversion to an autoantigen.

## References

[1] Bessman SP, Carpenter CL (1985) The creatine-creatine phosphate energy shuttle. Annu Rev Biochem 54: 831–862. 3896131 10.1146/annurev.bi.54.070185.004151

[2] McLeish MJ, Kenyon GL (2005) Relating structure to mechanism in creatine kinase. Crit Rev Biochem Mol Biol 40: 1–20. 15804623 10.1080/10409230590918577

[3] Wallimann T, Wyss M, Brdiczka D, Nicolay K, Eppenberger HM (1992) Intracellular compartmentation, structure and function of creatine kinase isoenzymes in tissues with high and fluctuating energy demands: the ‘phosphocreatine circuit’ for cellular energy homeostasis. Biochem J 281 (Pt 1): 21–40. 10.1042/bj2810021 PMC1130636 1731757

[4] Andres RH, Ducray AD, Schlattner U, Wallimann T, Widmer HR (2008) Functions and effects of creatine in the central nervous system. Brain Res Bull 76: 329–343. 18502307 10.1016/j.brainresbull.2008.02.035

[5] Jost CR, Van Der Zee CE, In 't Zandt HJ, Oerlemans F, Verheij M, et al (2002) Creatine kinase B-driven energy transfer in the brain is important for habituation and spatial learning behaviour, mossy fibre field size and determination of seizure susceptibility. Eur J of Neurosci 15: 1692–1706. 12059977 10.1046/j.1460-9568.2002.02001.x

[6] Aksenov M, Aksenova M, Butterfield DA, Markesbery WR (2000) Oxidative modification of creatine kinase BB in Alzheimer's disease brain. J Neurochem 74: 2520–2527. 10820214 10.1046/j.1471-4159.2000.0742520.x

[7] Nuss JE, Amaning JK, Bailey CE, DeFord JH, Dimayuga VL, et al (2009) Oxidative modification and aggregation of creatine kinase from aged mouse skeletal muscle. Aging (Albany NY) 1: 557–572. 20195383 10.18632/aging.100055 PMC2830079

[8] Burklen TS, Schlattner U, Homayouni R, Gough K, Rak M, et al (2006) The creatine kinase/creatine connection to Alzheimer's disease: CK-inactivation, APP-CK complexes and focal creatine deposits. J Biomed Biotechnol 2006: 35936. 17047305 10.1155/JBB/2006/35936 PMC1510941

[9] Zhu JX, Doyle HA, Mamula MJ, Aswad DW (2006) Protein repair in the brain, proteomic analysis of endogenous substrates for protein L-isoaspartyl methyltransferase in mouse brain. J Biol Chem 281: 33802–33813. 16959769 10.1074/jbc.M606958200

[10] Aswad DW, Paranandi MV, Schurter BT (2000) Isoaspartate in peptides and proteins: formation, significance, and analysis. J Pharm Biomed Anal 21: 1129–1136. 10708396 10.1016/s0731-7085(99)00230-7

[11] Clarke S (2003) Aging as war between chemical and biochemical processes: protein methylation and the recognition of age-damaged proteins for repair. Ageing Res Rev 2: 263–285. 12726775 10.1016/s1568-1637(03)00011-4

[12] Desrosiers RR, Fanelus I (2011) Damaged proteins bearing L-isoaspartyl residues and aging: a dynamic equilibrium between generation of isomerized forms and repair by PIMT. Curr Aging Sci 4: 8–18. 21204776

[13] Shimizu T, Matsuoka Y, Shirasawa T (2005) Biological significance of isoaspartate and its repair system. Biol Pharm Bull 28: 1590–1596. 16141521 10.1248/bpb.28.1590

[14] Mamula MJ, Gee RJ, Elliott JI, Sette A, Southwood S, et al (1999) Isoaspartyl post-translational modification triggers autoimmune responses to self-proteins. J Biol Chem 274: 22321–22327. 10428801 10.1074/jbc.274.32.22321

[15] Dai S, Ni W, Patananan AN, Clarke SG, Karger BL, et al (2013) Integrated proteomic analysis of major isoaspartyl-containing proteins in the urine of wild type and protein L-isoaspartate O-methyltransferase-deficient mice. Anal Chem 85: 2423–2430. 23327623 10.1021/ac303428h PMC3599293

[16] Cantor JR, Stone EM, Chantranupong L, Georgiou G (2009) The human asparaginase-like protein 1 hASRGL1 is an Ntn hydrolase with beta-aspartyl peptidase activity. Biochemistry 48: 11026–11031. 19839645 10.1021/bi901397h PMC2782781

[17] Patananan AN, Capri J, Whitelegge JP, Clarke SG (2014) Non-Repair Pathways for Minimizing Protein Isoaspartyl Damage in the Yeast Saccharomyces cerevisiae. J Biol Chem. 10.1074/jbc.M114.564385 PMC4059137 24764295

[18] Aswad DW (1984) Stoichiometric methylation of porcine adrenocorticotropin by protein carboxyl methyltransferase requires deamidation of asparagine 25. J Biol Chem 259: 10714–10721. 6088513

[19] Murray ED Jr, Clarke S (1984) Synthetic peptide substrates for the erythrocyte protein carboxyl methyltransferase. Detection of a new site of methylation at isomerized L-aspartyl residues. J Biol Chem 259: 10722–10732. 6469980

[20] Johnson BA, Langmack EL, Aswad DW (1987) Partial repair of deamidation-damaged calmodulin by protein carboxyl methyltransferase. J Biol Chem 262: 12283–12287. 3624258

[21] Brennan TV, Anderson JW, Jia Z, Waygood EB, Clarke S (1994) Repair of spontaneously deamidated HPr phosphocarrier protein catalyzed by the L-isoaspartate-(D-aspartate) O-methyltransferase. J Biol Chem 269: 24586–24595. 7929130

[22] Galletti P, Ciardiello A, Ingrosso D, Di Donato A (1988) Repair of isopeptide bonds by protein carboxyl O-methyltransferase: seminal ribonuclease as a model system. Biochemistry 27: 1752–1757. 3365422 10.1021/bi00405a055

[23] Reissner KJ, Paranandi MV, Luc TM, Doyle HA, Mamula MJ, et al (2006) Synapsin I is a major endogenous substrate for protein L-isoaspartyl methyltransferase in mammalian brain. J Biol Chem 281: 8389–8398. 16443604 10.1074/jbc.M510716200

[24] Johnson BA, Murray ED Jr, Clarke S, Glass DB, Aswad DW (1987) Protein carboxyl methyltransferase facilitates conversion of atypical L-isoaspartyl peptides to normal L-aspartyl peptides. J Biol Chem 262: 5622–5629. 3571226

[25] McFadden PN, Clarke S (1987) Conversion of isoaspartyl peptides to normal peptides: Implications for the cellular repair of damaged proteins. Proc Natl Acad Sci U S A 84: 2595–2599. 3472227 10.1073/pnas.84.9.2595 PMC304704

[26] Johnson BA, Najbauer J, Aswad DW (1993) Accumulation of substrates for protein L-isoaspartyl methyltransferase in adenosine dialdehyde-treated PC12 cells. J Biol Chem 268: 6174–6181. 8454593

[27] Kim E, Lowenson JD, MacLaren DC, Clarke S, Young SG (1997) Deficiency of a protein-repair enzyme results in the accumulation of altered proteins, retardation of growth, and fatal seizures in mice. Proc Natl Acad Sci U S A 94: 6132–6137. 9177182 10.1073/pnas.94.12.6132 PMC21014

[28] Yamamoto A, Takagi H, Kitamura D, Tatsuoka H, Nakano H, et al (1998) Deficiency in protein L-isoaspartyl methyltransferase results in a fatal progressive epilepsy. J Neurosci 18: 2063–2074. 9482793 10.1523/JNEUROSCI.18-06-02063.1998 PMC6792936

[29] Kosugi S, Furuchi T, Katane M, Sekine M, Shirasawa T, et al (2008) Suppression of protein l-isoaspartyl (d-aspartyl) methyltransferase results in hyperactivation of EGF-stimulated MEK-ERK signaling in cultured mammalian cells. Biochem Biophys Res Commun 371: 22–27. 18381200 10.1016/j.bbrc.2008.03.109

[30] Mizobuchi M, Murao K, Takeda R, Kakimoto Y (1994) Tissue-Specific Expression of Isoaspartyl Protein Carboxyl Methyltransferase Gene in Rat-Brain and Testis. J Neurochem 62: 322–328. 8263531 10.1046/j.1471-4159.1994.62010322.x

[31] Diliberto EJ Jr, Axelrod J (1976) Regional and subcellular distribution of protein carboxymethylase in brain and other tissues. J Neurochem 26: 1159–1165. 6627 10.1111/j.1471-4159.1976.tb07001.x

[32] Qin Z, Yang J, Klassen H, Aswad DW (2014) Isoaspartyl Protein Damage and Repair in Mouse Retina. Invest Ophthalmol Vis Sci. 10.1167/iovs.13-13668 PMC3954159 24550364

[33] Boivin D, Bilodeau D, Beliveau R (1995) Immunochemical characterization of L-isoaspartyl-protein carboxyl methyltransferase from mammalian tissues. Biochem J 309: 993–998. 7639720 10.1042/bj3090993 PMC1135729

[34] Ikegaya Y, Yamada M, Fukuda T, Kuroyanagi H, Shirasawa T, et al (2001) Aberrant synaptic transmission in the hippocampal CA3 region and cognitive deterioration in protein-repair enzyme-deficient mice. Hippocampus 11: 287–298. 11769310 10.1002/hipo.1043

[35] Vitali R, Clarke S (2004) Improved rotorod performance and hyperactivity in mice deficient in a protein repair methyltransferase. Behav Brain Res 153: 129–141. 15219714 10.1016/j.bbr.2003.11.007

[36] DeVry CG, Clarke S (1999) Polymorphic forms of the protein L-isoaspartate (D-aspartate) O-methyltransferase involved in the repair of age-damaged proteins. J Hum Genet 44: 275–288. 10496068 10.1007/s100380050161

[37] Chavous DA, Jackson FR, O'Connor CM (2001) Extension of the Drosophila lifespan by overexpression of a protein repair methyltransferase. Proc Natl Acad Sci U S A 98: 14814–14818. 11742076 10.1073/pnas.251446498 PMC64941

[38] Khare S, Gomez T, Linster CL, Clarke SG (2009) Defective responses to oxidative stress in protein l-isoaspartyl repair-deficient Caenorhabditis elegans. Mech Ageing Dev 130: 670–680. 19682488 10.1016/j.mad.2009.08.002 PMC2757507

[39] Steen C, Wilczak N, Hoogduin JM, Koch M, De Keyser J (2010) Reduced creatine kinase B activity in multiple sclerosis normal appearing white matter. Plos One 5: e10811. 20520825 10.1371/journal.pone.0010811 PMC2876025

[40] Khoury MK, Parker I, Aswad DW (2010) Acquisition of chemiluminescent signals from immunoblots with a digital single-lens reflex camera. Anal Biochem 397: 129–131. 19788886 10.1016/j.ab.2009.09.041 PMC2808431

[41] Macfarlane DE (1984) Inhibitors of cyclic nucleotide phosphodieterases inhibit protein carboxyl methylation in intact blood platelets. J Biol Chem 259: 1357–1362. 6198323

[42] Johnson BA, Shirokawa JM, Geddes JW, Choi BH, Kim RC, et al (1991) Protein L-isoaspartyl methyltransferase in postmortem brains of aged humans. Neurobiol Aging 12: 19–24. 2002878 10.1016/0197-4580(91)90034-h

[43] Johnson BA, Aswad DW (1991) Optimal conditions for the use of protein L-isoaspartyl methyltransferase in assessing the isoaspartate content of peptides and proteins. Anal Biochem 192: 384–391. 1827964 10.1016/0003-2697(91)90553-6

[44] Liu M, Cheetham J, Cauchon N, Ostovic J, Ni W, et al (2012) Protein isoaspartate methyltransferase-mediated 18O-labeling of isoaspartic acid for mass spectrometry analysis. Anal Chem 84: 1056–1062. 22132761 10.1021/ac202652z

[45] Paranandi MV, Guzzetta AW, Hancock WS, Aswad DW (1994) Deamidation and isoaspartate formation during in vitro aging of recombinant tissue plasminogen activator. J Biol Chem 269: 243–253. 8276801

[46] Carter WG, Aswad DW (2008) Formation, localization, and repair of L-isoaspartyl sites in histones H2A and H2B in nucleosomes from rat liver and chicken erythrocytes. Biochemistry 47: 10757–10764. 18795804 10.1021/bi8013467

[47] Qin Z, Kaufman RS, Khoury RN, Khoury MK, Aswad DW (2013) Isoaspartate accumulation in mouse brain is associated with altered patterns of protein phosphorylation and acetylation, some of which are highly sex-dependent. PLoS One 8: e80758. 24224061 10.1371/journal.pone.0080758 PMC3818261

[48] Jazin E, Cahill L (2010) Sex differences in molecular neuroscience: from fruit flies to humans. Nat Rev Neurosci 11: 9–17. 20019686 10.1038/nrn2754

[49] Young GW, Hoofring SA, Mamula MJ, Doyle HA, Bunick GJ, et al (2005) Protein L-isoaspartyl methyltransferase catalyzes in vivo racemization of Aspartate-25 in mammalian histone H2B. J Biol Chem 280: 26094–26098. 15908425 10.1074/jbc.M503624200

[50] Johnson BA, Shirokawa JM, Hancock WS, Spellman MW, Basa LJ, et al (1989) Formation of isoaspartate at two distinct sites during in vitro aging of human growth hormone. J Biol Chem 264: 14262–14271. 2760065

[51] Eder M, Schlattner U, Becker A, Wallimann T, Kabsch W, et al (1999) Crystal structure of brain-type creatine kinase at 1.41 A resolution. Protein Sci 8: 2258–2269. 10595529 10.1110/ps.8.11.2258 PMC2144193

[52] Olsen JV, Vermeulen M, Santamaria A, Kumar C, Miller ML, et al (2010) Quantitative phosphoproteomics reveals widespread full phosphorylation site occupancy during mitosis. Sci Signal 3: ra3. 20068231 10.1126/scisignal.2000475

[53] Tetsuka S, Tominaga K, Ohta E, Kuroiwa K, Sakashita E, et al (2013) Paraneoplastic cerebellar degeneration associated with an onconeural antibody against creatine kinase, brain-type. J Neurol Sci 335: 48–57. 24018129 10.1016/j.jns.2013.08.022

[54] Zhu L, Shen W, Zhu M, Coorey NJ, Nguyen AP, et al (2013) Anti-retinal antibodies in patients with macular telangiectasia type 2. Invest Ophthalmol Vis Sci 54: 5675–5683. 23882694 10.1167/iovs.13-12050

[55] O'Connor CM, Yutzey KE (1988) Enhanced carboxyl methylation of membrane-associated hemoglobin in human erythrocytes. J Biol Chem 263: 1386–1390. 3335550

[56] Ingrosso D, D'Angelo S, di Carlo E, Perna AF, Zappia V, et al (2000) Increased methyl esterification of altered aspartyl residues in erythrocyte membrane proteins in response to oxidative stress. Eur J Biochem 267: 4397–4405. 10880963 10.1046/j.1432-1327.2000.01485.x

[57] D'Angelo S, Ingrosso D, Migliardi V, Sorrentino A, Donnarumma G, et al (2005) Hydroxytyrosol, a natural antioxidant from olive oil, prevents protein damage induced by long-wave ultraviolet radiation in melanoma cells. Free Rad Biol Med 38: 908–919. 15749387 10.1016/j.freeradbiomed.2004.12.015

[58] Curnis F, Longhi R, Crippa L, Cattaneo A, Dondossola E, et al (2006) Spontaneous formation of L-isoaspartate and gain of function in fibronectin. J Biol Chem 281: 36466–36476. 17015452 10.1074/jbc.M604812200

[59] Barbariga M, Curnis F, Spitaleri A, Andolfo A, Zucchelli C, et al (2014) Oxidation-induced structural changes of ceruloplasmin foster NGR motif deamidation that promotes integrin binding and signaling. J Biol Chem 289: 3736–3748. 24366863 10.1074/jbc.M113.520981 PMC3916571

[60] Cimmino A, Capasso R, Muller F, Sambri I, Masella L, et al (2008) Protein isoaspartate methyltransferase prevents apoptosis induced by oxidative stress in endothelial cells: role of Bcl-Xl deamidation and methylation. PLoS One 3: e3258. 18806875 10.1371/journal.pone.0003258 PMC2532751

[61] Eliuk SM, Renfrow MB, Shonsey EM, Barnes S, Kim H (2007) active site modifications of the brain isoform of creatine kinase by 4-hydroxy-2-nonenal correlate with reduced enzyme activity: mapping of modified sites by Fourier transform-ion cyclotron resonance mass spectrometry. Chem Res Toxicol 20: 1260–1268. 17696488 10.1021/tx7000948

[62] Okunuki Y, Usui Y, Kezuka T, Hattori T, Masuko K, et al (2008) Proteomic surveillance of retinal autoantigens in endogenous uveitis: implication of esterase D and brain-type creatine kinase as novel autoantigens. Mol Vis 14: 1094–1104. 18552983 PMC2426731

[63] Doyle HA, Gee RJ, Mamula MJ (2007) Altered immunogenicity of isoaspartate containing proteins. Autoimmunity 40: 131–137. 17453712 10.1080/08916930601165180

