# Correction: Isoaspartyl Formation in Creatine Kinase B Is Associated with Loss of Enzymatic Activity; Implications for the Linkage of Isoaspartate Accumulation and Neurological Dysfunction in the PIMT Knockout Mouse

**DOI:** 10.1371/journal.pone.0116556

**Published:** 2014-12-22

**Authors:** The PLOS ONE Staff

There is an error in the last sentence of the “Mice” subsection of the Materials and Methods. The correct sentence is: Mice were anesthetized with a lethal dose of Euthasol prior to decapitation at 4-5 weeks of age.

There is an error in the first sentence of the “Preparation of Mouse Brain Extracts” subsection of the Materials and Methods. The correct sentence is: Mouse brains were weighed immediately after removal and suspended in 9 vol of ice-cold homogenization buffer (10% (w/v) sucrose, 5 mM K-Hepes, pH 7.6, 0.5 mM EDTA, 0.1 mM DTT (dithiothreitol), 50 mM NaF and 1 mM Na_3_VO_4_ as phosphatase inhibitors, and 1% (v/v) mammalian protease inhibitor mixture (Sigma).

There is an error in the last sentence of the “Protein Concentration and Enzyme Activity” subsection of the Materials and Methods. The correct sentence is: *One unit of CKB activity is defined here as one µmol of NADPH generated per min at 25°C under initial rate conditions.*
